# Investigating the structure of semantic networks in low and high creative persons

**DOI:** 10.3389/fnhum.2014.00407

**Published:** 2014-06-10

**Authors:** Yoed N. Kenett, David Anaki, Miriam Faust

**Affiliations:** ^1^The Leslie and Susan Gonda (Goldschmied) Multidisciplinary Gonda Brain Research Center, Bar-Ilan UniversityRamat-Gan, Israel; ^2^Department of Psychology, Bar-Ilan UniversityRamat-Gan, Israel

**Keywords:** creativity, associative thinking, network science, individual differences, semantic networks

## Abstract

According to Mednick's (1962) theory of individual differences in creativity, creative individuals appear to have a richer and more flexible associative network than less creative individuals. Thus, creative individuals are characterized by “flat” (broader associations) instead of “steep” (few, common associations) associational hierarchies. To study these differences, we implement a novel computational approach to the study of semantic networks, through the analysis of free associations. The core notion of our method is that concepts in the network are related to each other by their association correlations—overlap of similar associative responses (“association clouds”). We began by collecting a large sample of participants who underwent several creativity measurements and used a decision tree approach to divide the sample into low and high creative groups. Next, each group underwent a free association generation paradigm which allowed us to construct and analyze the semantic networks of both groups. Comparison of the semantic memory networks of persons with low creative ability and persons with high creative ability revealed differences between the two networks. The semantic memory network of persons with low creative ability seems to be more rigid, compared to the network of persons with high creative ability, in the sense that it is more spread out and breaks apart into more sub-parts. We discuss how our findings are in accord and extend Mednick's (1962) theory and the feasibility of using network science paradigms to investigate high level cognition.

## Introduction

Creativity is one of the few qualities that define human nature (Lindell, 2010). While in the past, the mental processes enabling creativity were considered mystical and un-researchable, nowadays an ample body of research has been established, permitting the examination of the creative ability like any other cognitive process (Dietrich, 2004; Dietrich and Kanso, 2010; Sawyer, 2011; Abraham, 2013). Dietrich (2004) argues that creativity is not a special feature of the cognitive system, but rather requires a variety of classic cognitive abilities such as working memory, sustained attention, and cognitive flexibility. Thus, this perspective allows breaking down the concept of creativity into specific cognitive abilities which can be measured separately with conventional empirical measures. One of the areas that have been extensively studied within the multifaceted concept of creativity is linguistic semantic creativity. Semantic creativity refers to flexibility, fluency and originality which results in high-order language products such as irony, humor, and metaphors (Faust, 2012; Mirous and Beeman, 2012). Such high-order language products share the need of the language system to process and maintain multiple alternative meanings of a concept, including meanings which are distantly or unusually connected (Cushen and Wiley, 2011). As such, semantic creativity is realized by the association of seemingly unrelated or distantly related concepts that nevertheless create a meaningful linguistic expression. Hence, the semantic network of semantically creative persons may be different than that of less creative people, allowing for more flexible and novel conceptual combinations during semantic processing. The goal of the present research is to quantitatively examine individual differences in the semantic networks of individuals with low semantic creative (LSC) and high semantic creative (HSC) abilities.

One of the hallmarks of creativity is that memory is searched more widely and in a less-defined manner than during everyday thinking (Bink and Marsh, 2000; Lindell, 2010). Thus, when describing creativity, Amabile et al. (2005) and Simonton (1999) note that the larger the number of potentially relevant elements that are retrieved during processing, the higher the likelihood that unusual associations or solutions will be generated, and the larger is the pool of novel ideas from which to choose. Recent reviews on creative thinking support this claim by emphasizing the retrieval of remote associations during creative problem solving (Helie and Sun, 2010). This view was strengthened by Friedman and Förster (2002) who presented empirical evidence that creative behavior can be mediated by a memory search-based mechanism. Finally, Griffiths et al. (2007) provide theoretical and empirical evidence to the similarity between memory search and the Google search engine algorithm. Such search processes are executed while people engage in semantic creativity tasks, and is the basis of Mednick's (1962) theory of creativity and his Remote Association Test (RAT).

Mednick (1962), focusing on individual differences in semantic creativity, envisioned the creative process as the combination of remote associations into new and useful combinations. To examine his theory, Mednick developed the RAT. In this test, subjects are presented with a triplet of seemingly unrelated words (e.g., *Cottage, Swiss, Cake*) and are required to find a single fourth word that is related to each of these words (e.g., Cheese; Bowden and Jung-Beeman, 2003). This task is accepted as examining semantic creativity and has been empirically widely used for its investigation (Gold et al., 2011; Storm et al., 2011; Mirous and Beeman, 2012). Investigating the significance of combination of remote associations, Benedek et al., recently demonstrated the importance of the RAT in predicting divergent thinking (the hallmark factor of creative ability) and intelligence (Benedek et al., 2012b). Thus, creative ability is highly related to associative thinking, a notion that has recently been corroborated in a critical review of neurocognitive research on creativity (Sawyer, 2011).

Despite the wide use of the RAT to investigate creativity, some argue against the RAT as a measure of creativity (Taft and Rossiter, 1966; Lee and Therriault, 2013). These objections are mainly due to the fact that the RAT is considered a convergent, and not a divergent, measure of creativity. Divergent thinking refers to an ideational process which involves generating a broad range of solutions or ideas to a given stimuli and is considered the hallmark of creative ability (Runco and Acar, 2012; Lee and Therriault, 2013). Convergent thinking, on the other hand, is considered a deductive process that involves systematically applying rules to arrive at a single, correct solution (Brophy, 2001; Lee and Therriault, 2013). As the RAT measures the success of a participant to find the single correct solution, it is considered a convergent test of creativity. However, Taft and Rossiter (1966) examined whether the RAT measures divergent or convergent modes of thought, by having participants complete the RAT with other convergent (such as school achievement and verbal IQ) and divergent (such as ideational and word fluency) measures. While the authors show how the RAT highly correlated with the convergent measures (measured by IQ and achievement scores), they also found significant correlations between the RAT and the divergent measures (measured by flexibility, originality, and fluency scores). Thus, it can be concluded from the work of Taft and Rossiter (1966) that the RAT demands both convergent and divergent thinking.

Recent studies examine performance in the RAT from a cognitive search perspective (Gupta et al., 2012; Smith et al., 2013). Smith et al. (2013) view the RAT as a multiple constraint problem, in which each cue word indicates a different attribute of the target word. Solving such a multiple constrained problem requires a two stage process: first, a search for a possible solution is conducted and then this candidate solution is tested against all of the constrains of the problem to rate the acceptability of the solution (Smith et al., 2013). Smith et al. (2013) found that participants solve RAT problems first by selecting a set of possible answers constrained by a single cue word at a time. Furthermore, the authors show how prior candidate answers directly affect the following guesses, suggesting an associatively connected directed search, which is in agreements with the spreading activation model (Collins and Loftus, 1975). By examining the guesses provided by participants in attempting to solve RAT problems, the authors focus on the search process required in the RAT and not the end solution. This perspective may resolve the convergent-divergent debate of the RAT. As Smith et al. (2013) show, the RAT first requires a divergent thinking process to generate candidate solutions and then executive functions are required to examine the acceptability of the solution (see also Klein and Badia, 2014). Thus, the differences between low and high creative persons can be related to the structure of their semantic memory, executive functions, or both. Here we will focus on any possible differences related to the structure of semantic memory.

Mednick's (1962) theory of individual differences in associative hierarchies proposes that creative individuals have a richer and more flexible associative network than less creative individuals. According to his theory (Mednick, 1962), creative individuals are characterized by “flat” (more and broader associations to a given stimulus) instead of “steep” associational hierarchies few, common associations to a given stimulus (but see Benedek and Neubauer, 2013 for an opposing view). Thus, creative individuals may have more associative links in their network and can connect associative relations faster than less creative individuals, thereby facilitating more efficient search processes (Rossman and Fink, 2010). Gruszka and Necka (2002) examined the priming of close and remote associations by low creative and high creative persons. They show how high creative participants may be characterized by having a more complex lexicon network structure and how high creative participants may activate a wider range of associations across their lexicon network (Gruszka and Necka, 2002). Rossman and Fink (2010) found that creative subjects give lower estimates of the semantic distance between unrelated word pairs as compared to less creative subjects, implying that the former group may have a wider, interconnected semantic network which could lead to more efficient search process compared to less creative persons. To date, no direct examination of the difference in semantic network organization between low and high creative persons exists. Such examination of semantic memory networks has been recently become possible through the use of network science tools.

Semantic memory is the system of human memory that is responsible for the storage of semantic categories and of natural and artificial concepts (Budson and Price, 2005; Patterson et al., 2007). However, the way in which semantic memory is organized into categories and subcategories remains an open question (Rogers, 2008). Recently, this issue is more and more directly addressed via the application of computational network tools. Network science is based on mathematical graph theory, providing quantitative methods to investigate complex systems as networks. A network is comprised from nodes, which represent the basic unit of the system (e.g., mental lexicon) and links, or edges, that signify the relations between them (e.g., semantic similarity). This field has greatly advanced in the past few decades due to technological and quantitative theoretical advances, which allowed a rapid development of tools and theory to investigate both structural properties and dynamics of a network (reviewed in Baronchelli et al., 2013). Of the various network models developed in network science theory, the network model that has been widely used to examine complex systems is the Small World Network model (SWN; Milgram, 1967; Watts and Strogatz, 1998). This model has successfully described a wide range of sociological, technological, biological and economical networks (Boccaletti et al., 2006; Cohen and Havlin, 2010; Kenett et al., 2010; Newman, 2010) and is also widely used in studying structural and functional brain networks (Sporns, 2011; Bullmore and Sporns, 2012; Stam and van Straaten, 2012; van Straaten and Stam, 2013).

Two main characteristics of SWN are the networks clustering coefficient (CC) and its average shortest path length (ASPL). The CC refers to the probability that two neighbors (a neighbor is a node *j* that is connected through an edge to node *i*) of a node will themselves be neighbors. The ASPL refers to the average shortest amount of steps (nodes being traversed) needed to be taken between any two pair of nodes. A SWN is characterized by having a large CC and a short ASPL. Further structural properties of such network are the network diameter (D), which represents the largest path length in the network (and thus related to the spread of the network). Furthermore, network science examines the community structure of complex networks (Fortunato, 2010). Community structure research examines how a complex system, comprised of many nodes and edges, break apart (or partition) into smaller sub-networks. This area of research has been promoted, to a great extent, by Newman (2006), who introduced the notion of Modularity (Q). The modularity measure is a statistical measure that quantifies how much a network is partitioned into sub-communities. The larger the modularity measure is, the more the network is comprised from sub communities (Newman, 2006). The notion of modularity is extensively investigated at the neural (Meunier et al., 2010; Bullmore and Sporns, 2012; Hilgetag and Hütt, 2014), and more recently, cognitive (Arenas et al., 2012; Kenett et al., under review) levels of brain organization. Finally, a recent measure has been presented (S), which aims to quantitatively measure the “small-world-ness” feature of a specific network (Humphries and Gurney, 2008). This measure is a ratio of the CC and ASPL and allows investigating how much a network is “small-worlded,” to the extent that any S-value greater than one is a SWN. In order to examine the small-world nature of an empirical network, its statistical properties are compared to those of a random, null network with the same amount of nodes and edges (Boccaletti et al., 2006).

At the cognitive level (the level of information processing in the brain), application of network science tools is also developing, mainly to investigate complex systems of language and memory structure (Vitevitch, 2008; Borge-Holthoefer and Arenas, 2010; Chan and Vitevitch, 2010; Vitevitch et al., 2012, 2014; Baronchelli et al., 2013). In the linguistic domain, lexicons of different languages seem to display SWN characteristics, considered to be a fundamental principle in lexical organization (Steyvers and Tenenbaum, 2005; De-Deyne and Storms, 2008a,b; Borge-Holthoefer and Arenas, 2010; Kenett et al., 2011). Investigating the complexity of semantic knowledge with network science allows to uniquely examine fundamental questions such as the nature of semantic organization (what are the structural principles that characterize semantic knowledge?), process and performance (to what extent can human performance in semantic processing tasks be explained in terms of general processing in semantic memory network?) and typical and non-typical semantic lexicon development (Steyvers and Tenenbaum, 2005; Beckage et al., 2011; Kenett et al., 2013). In fact, network research in language is slowly shifting from an interest in investigating the structure of mental lexicons to investigating cognitive processes operating on these lexicon networks (Borge-Holthoefer and Arenas, 2010; Arenas et al., 2012). We have recently introduced a novel approach to the study of semantic networks (Kenett et al., 2011) that makes use of correlation and network methodologies to define semantic similarity between concepts in the semantic network. The core idea of our method is the definition of connections between concepts in the semantic network by the similarity of association responses generated to these concepts, or alternatively, as the overlap of “association clouds.” This notion is in accord with classic cognitive theory on the organization of semantic memory (Collins and Loftus, 1975), and thus differs from standard methods of extracting semantic similarity based on standard statistical properties (Kenett et al., 2011). Thus, such a method is suitable to study the differences between low and high creative persons, as proposed by Mednick (1962), which are theoretically found in their structure of associative hierarchies.

A small but slowly growing amount of research investigating creativity with network science tools is starting to appear. Schilling (2005) has presented a theory that suggests that insight problem solving is a result of a successful search throughout semantic memory network, enabled by either finding “shortcuts” or by the creation of new links between previously unconnected nodes in the network. Yet, this theory has not been empirically examined. Kenett et al. (2011) proposed that the structure of the mental lexicon constrains cognitive search processes such as those required in the RAT. Recently, a neural network model has been proposed aiming to model the dynamics of spontaneous thought (the spontaneous emergence of ideas), by directly examining associative processes such as those in the RAT (Marupaka et al., 2012). The basic assumptions of this model are that all thought is homogeneous, combinatorial and associative, which converge with Mednick's (1962) theory of creativity. At the core of this model lies the idea of a neural semantic network—concepts in semantic memory are somehow organized together, and this structure allows spontaneous thought to occur (for more details, see Marupaka et al., 2012). The authors examine various types of network models which account for different organization of semantic memory, and conclude that the best model to describe semantic memory is the SWN model. Recently, Doumit et al. used this model to analyze the writings of prominent poets (i.e., Dylan Thomas) and writers (i.e., F. Scott Fitzgerald), by extracting their associative networks based on their textual corpora which contain a varying degree of creative language (Doumit et al., 2013). This was done to investigate whether their neural network model can account for the difference in associative networks of “more creative” poetic texts vs. “less creative,” more structured, prosaic texts. The authors show that the “more creative” poet corpora exhibited a “flatter” associative distribution than the “less creative” prose corpora (see Doumit et al., 2013). Nevertheless, as the authors admit themselves, this work is quite preliminary and requires further investigation. Furthermore, both corpora analyzed in this research are comprised from skilled and creative individuals (either poets or prose writers).

In the present research, we apply a network science methodology to directly and quantitatively examine Mednick's (1962) theory of individual differences in low and high creative persons. We collected a large sample of participants who underwent several creativity measures and were divided into a LSC and HSC groups. The approach developed by Kenett et al. (2011) was used to represent and compare the semantic networks of both groups. First, LSC and HSC groups generated free associations to 96 target words. Next, the semantic networks of both groups were calculated based on the overlap of association responses (“associative clouds”) between the target words. Finally, we quantitatively analyzed and compared the two networks to examine any possible difference between them. We hypothesized, in accordance with Mednick's (1962) theory, that the LSC network would be more modular than the HSC network (higher Q measure for the LSC network). Furthermore, in accordance with Rossman and Fink (2010) findings, we hypothesized that the LSC network would be less condensed than the HSC network (higher ASPL and D measures for the LSC network). Finally, in accordance with Schilling's (2005) theory, we expected the LSC network to be less connected than the HSC network (lower CC and S measures for the LSC network).

## Methods

### Participants

One hundred and forty-four persons were recruited for the study. Five subjects were removed from the final sample (three subjects due to incompliance with the tasks and the data of two subjects were lost due to technical issues), resulting in a final sample of 139 subjects (47 men, 92 women), with mean age of 23 years (*SD* = 2.4). All subjects were Hebrew native speakers, had normal or corrected to normal eyesight and were right handed, as measured by the Edinburgh Handedness Inventory (EHI; Oldfield, 1971; mean score = 92, *SD* = 9). Subjects either took part in the study as partial fulfillment of academic credit or were paid for their participation. This experiment was approved by the Bar-Ilan University institutional review board.

### Materials

#### Creativity measurement

***Remote Association Test***. The RAT (Mednick, 1962) was developed to investigate individual differences in creative ability (as described above). In our research we used the Hebrew version of the RAT (Nevo and Levin, 1978) which contains 25 triplets with varying degree of difficulty and lasts 15 min. The RAT score is the sum of correct answers given by the participant.

***Tel-Aviv University Creativity Test (TACT; Milgram and Milgram, 1976)***. This test is a modified Hebrew version of the Wallach and Kogan (1965) battery of creativity tests (see Kaufman et al., 2012 for a current review of creativity measurements). This battery of tests includes several different measures of divergent thinking, which is considered the hallmark predictor of creative ability (Runco and Acar, 2012), frequently used in creativity research (Baird et al., 2012). The TACT measures verbal and visual creativity by producing two scores—fluency (number of responses provided), and quality (originality and applicability of response). The test is comprised of four sub-tests—two verbal (alternative uses and pattern matching) and two visual (similarities and line meanings). Each sub-test lasts 6 min and includes four open questions. The results of both verbal and visual sub-tests of the TACT were combined into TACT verbal and TACT visual scores. Fluency score was calculated by counting the number of different answers, and quality score was determined by three independent judges judging the originality and applicability of responses to stimuli for unique answers only, namely, answers which appeared in only 5% or less of the sample (Milgram and Milgram, 1976).

***Comprehension of Metaphors (CoM; Faust, 2012)***. In this task, subjects are presented with word-pairs in Hebrew, which can either have a literal, conventional metaphoric, novel metaphoric meaning or are meaningless, and are asked to decide whether the two words comprise a semantically meaningful expression or not (Faust, 2012). This paradigm has been used in converging behavioral and neurocognitive techniques to investigate neural and hemispheric processing of novel metaphors compared to conventional metaphors, literal expressions and unrelated, meaningless word-pairs (reviewed in Faust, 2012). Recently, a significant positive correlation between scores on this on-line semantic judgment task for processing novel metaphors and the RAT has been shown (Gold et al., 2011). As such, this semantic judgment task provides a further measure of semantic creative ability (see also Silvia and Beaty, 2012).

***Raven Progressive Matrices Test-Short Version (RSPM-SV; Van der Elst et al., 2013)***. In order to rule out any artifacts due to intelligence (Silvia and Beaty, 2012; Lee and Therriault, 2013), all participants underwent the Raven progressive matrices test (Raven and Raven, 2008). We used the shorten version of the RSPM, which has recently been shown by Van der Elst et al. (2013) to be a short valid method to assess intelligence, while taking into consideration age and gender effects on RSPM performance. This shortened version includes only series B, C and D of the original RSPM (Van der Elst et al., 2013).

#### Classifying participants into LSC and HSC groups

The TACT battery of creativity measures was used to classify the participants into LSC and HSC groups. One possible way to do so is to divide the sample into quarters, or thirds and compare the lowest quarter (or third) against the top quarter (or third) (Altman and Bland, 1994). However, recent objections have been raised at this method, especially when measuring continuous variables such as creative ability (Preacher et al., 2005). Preacher et al. (2005) discuss several challenges of what they term the “extreme groups analysis,” related to statistical power, effect size, and group selection (see Preacher et al., 2005). Such concerns call for a more objective method to classify the participants into LSC and HSC groups. In this research, we used the decision tree approach, which is a statistical method at analyzing multivariate data (Lafond et al., 2009; Galimberti and Soffritti, 2011; Brandmaier et al., 2013). This approach has been mainly used in medicine and biology and is now being applied in psychological research, among other applications to classify subjects into low and high groups (Kopiez et al., 2006; Lafond et al., 2009; Strobl et al., 2009). Kopiez et al. (2006) used this approach to classify participants into low and high musical “sight-reading” ability (i.e., unrehearsed performance of music) groups, based on multiple independent variables. Thus, the decision tree approach is an efficient approach when analyzing a construct which has no comprehensive model to classify into low and high ability groups, such as creativity (Runco and Jaeger, 2012). Decision trees are implemented by a family of statistical algorithms that identify ways to split a multidimensional dataset into branch like segments (deVille, 2006). A decision tree attempts to predict, based on independent variables (for example, different measures of the TACT) specific classes of a dependent variable (for example, all participants who received a certain score on the RAT). The dependent variable can be split into smaller and smaller classes (branches), till specific stopping rules are achieved (Galimberti and Soffritti, 2011; Brandmaier et al., 2013). Thus, this method strives to find clusters that represent a sufficient range of the dependent variable and are separable with an accepted error (Kopiez et al., 2006). This method derives decisions, or classification rules, which form the different branches of the tree. Such rules are based on a method that extracts the relationship between the classes of the dependent variable and certain aspects of the independent variables (i.e., range of values in one specific variable and another range of another variable). The values in the independent variables are used to estimate the likely value in a specific class of the dependent variable. Once the relationship is extracted, one or more decision rules can be derived that describe the relationships between the independent variables and classes of the dependent variable.

In our research, we used the divergent thinking measures (TACT scores) as the independent variables and the participants' RAT scores as the dependent variable. This was chosen since divergent thinking is widely accepted as a measure of creativity (Runco and Acar, 2012) and the RAT measures another aspect of creativity, namely convergent thinking, in addition to divergent thinking, as discussed above. Thus, we reasoned that classifying the participants by the ability of their divergent thinking scores to estimate their RAT scores will result in a valid and reliable classification. In this sense, the decision tree classifier aims to predict RAT scores via various TACT measures classification rules. In this sense, this approach attempts to classify the participants based on their TACT performance into each of the possible RAT scores (1–25) and thus objectively sort participants into a low creative and a high creative sample. Finally, we will verify the validity of this classification method by examining the difference in performance of the two groups on the CoM task, which has been shown to measure creative ability (Gold et al., 2011; Silvia and Beaty, 2012).

#### Free association task

The free association task is based on the method used in Rubinstein et al. (2005), where subjects are presented with a target word and have one minute to generate as many associative responses they could for that target word. This method differs from classical association tasks, where subjects are only required to generate either one or three associative response to a target word (Nelson et al., 2004; De-Deyne and Storms, 2008a). This method is superior to previous methods in collecting association norms, as it exposes a greater part of the mental lexicon, helping to statistically strengthen significant associations to target words within the network (Kenett et al., 2011; De-Deyne et al., 2013).

The target words used in the free associations task were taken from Kenett et al. (under review). These words were drawn from a list of 36 categorical norms gathered by Henik and Kaplan (2005; e.g., fruits, trees, countries). The top 4 high frequency words from each category were selected. These high frequency words were then tested for their degree of concreteness by independent judges. Only words which were judged to be concrete were selected. The final target word pool thus consisted of 96 words from 24 categories (Kenett et al., under review).

#### Association correlation networks

The association correlation matrix is computed from the association data. The correlations between the target word associations profiles (the associative responses given to the target words by all subjects), are calculated by Pearson's correlation. This correlation is based on the contribution of two parameters—the extent of similar associative responses given to a pair of target words and the amount of participants generating these similar associative responses to these target words. Thus, the more similar associations generated and the larger amount of participants generating these association responses to a pair of target words, the higher the association correlation between this pair of words is. The target word-target word correlations (or for simplicity association correlations) for all pairs of words define a symmetric correlation matrix whose (*i, j*) element is the correlation between target words *i* and *j*.

For example, if a pair of target words are *dad* and *mom* we examine the overlap of associative responses for these two target words. A possible overlap of associative responses given both to the target word *dad* and the target word *mom* can be *family* (given by *a* amount of participants to *dad* and *b* amount of participants to *mom*), *home* (given by *c* amount of participants to *dad* and *d* amount of participants to *mom*), *love* (given by *e* amount of participants to *dad* and *f* amount of participants to *mom*) and so on. Then, each of the associative responses given to both target words and the amount of participants generating these associative responses for both target words is taken into account, in relation to all of the associative responses generated to each of the two target words and their standard deviation, to generate an association correlation between the two target words. Note that the association correlation was determined on the basis of the overlap of targets' responses. If a target words was generated as a response it was not included in the computation of the association correlation between these two target words.

The association correlation matrix can be studied in terms of an adjacency matrix of a weighted, undirected network. In this view, each target word is a node in the network, and an edge (link) between two nodes (words) is the association correlation between them, with the correlation value being the weight of that link. Since most of the edges have small values (weak correlations), the relevant information about the network can be obscured. To overcome this obstacle, we make use of the Planar Maximally Filtered Graph (PMFG; Tumminello et al., 2005) to construct from the complete network a sub-graph that captures the most relevant information embedded in the original network. This method is based on hierarchical clustering and the resulting sub-graph includes all the nodes in the network whose edges represent the most relevant association correlations. To construct the PMFG the *N*(*N* − 1) values of the correlation matrix are ordered in decreasing rank. The method starts from the pair of nodes *i* and *j*, with the highest correlation and draws a link *j*→*i* between them. This reiterates according to rank order where in each iteration, a link is added if and only if is the resulting graph is still planar, i.e., can be drawn on the surface of a sphere without link crossing (Tumminello et al., 2005). Since we are interested in the structure of the semantic networks, we binarized each association correlation network (by converting all edges to uniform weight = 1) and analyzed these networks as unweighted undirected networks.

Finally, it is important to note that this method can only examine group sample networks and is not sensitive to individual differences of specific participants (see Morais et al., 2013 for a novel approach measuring individual semantic networks). When applying this method to compare between two networks (for example, LSC vs. HSC semantic networks), this method focuses on how the responses generated to all target words in one group differ from that of the second group. Thus, if the same target words are presented to both groups in a free association task, this computational method analyzes the general difference of the network structure arising from each complete sample.

#### Network analysis

To empirically analyze and compare the structural network properties of the LSC and HSC semantic networks, the nodes in both networks must be controlled in order to eliminate any possible spurious results (van Wijk et al., 2010). This was achieved by constraining both networks to 96 target words. We did not control for the number of edges in the two networks. Network parameters calculated, with the MatLab Brain Connectivity Toolbox (Rubinov and Sporns, 2010) were: CC, ASPL, the average mean amount of edges per node [<k>, van Wijk et al., 2010 and the network's diameter (D)]. Furthermore, in order to examine the network's CC and ASPL, a random network was created with the same number of nodes and edges. For this random network, we calculated its clustering coefficient (CCrand) and its average shortest path length (ASPLrand). To examine the modularity of each network, we made use of Newman's modularity measure (Newman, 2006) to investigate how each network divides into sub-clusters of words, by calculating its modularity index (Q). Finally, the S measure (Humphries and Gurney, 2008) was computed to quantitatively evaluate the small-world nature of each network.

We also investigated the importance of each node in the network. In network theory, the importance of a node in a given network is quantified using different measures, such as the betweeness measure and eigenvalue centrality (Boccaletti et al., 2006). Here we used the word centrality measure (Kenett et al., 2011). The impact of a specific node is quantified as the difference between the ASPL of the network after removing word *i* with the ASPL of the full network. A positive impact score signifies that after the deletion of word *i*, the ASPL became longer than the ASPL of the full network, indicating that this word has a positive effect on the spread of activation within the network. We refer to these words as “facilitating nodes” (FN). In contrast, a negative impact score signifies that after the deletion of word *i*, the ASPL became shorter than the ASPL of the full network, indicating that this word has a negative effect on the spread of activation within the network. We refer to these words as “inhibiting nodes” (IN). this method allows us to investigate the effect each node has on the spread of activation in the network (see Vitevitch and Goldstein, 2014 for a similar approach).

Statistical hypothesis testing methods to compare between networks is currently lacking (Moreno and Neville, 2013). Such methods are required when conducting empirical network research to determine whether two (or more) networks are significantly different from each other or not (null hypothesis). This lack of network comparison hypothesis testing is mainly due to difficulties in estimating or collecting a large sample of empirical networks and only few statistical methods to compare between networks (see Moreno and Neville, 2013). To statistically analyze our findings, we used three complementing approaches. First, we simulated random networks to determine that the network measures calculated for both networks did not result from a null-hypothesis of a random network. To this end, we generated a large sample of Erdos-Renyi random networks with a fixed edge probability (Boccaletti et al., 2006) and compared the network measures to the values resulting from the simulated random distributions for each measure. Second, we examined whether differences between the LSC and HSC network measures were statistically significant by applying the bootstrap method (Efron, 1979) to simulate partial random LSC and HSC networks and compared these networks. This procedure had a twofold rationale: (1) if the two networks truly differ from each other, then any sub-network consisting of the same nodes in both networks should also be different, and (2) the bootstrap method enables the generation of many simulated partial LSC and HSC networks, allowing for statistical examination of the difference between the two networks. In order to conduct the bootstrapping procedure, half of the target words (nodes) were randomly chosen. Then partial LSC and HSC networks were constructed separately using these random nodes, and for each partial LSC and HSC network, CC, L, S, and Q measures were computed. This procedure was simulated with 1000 realizations. Finally, we analyzed the difference in the amount of unique association generation per target word between the two groups. If the LSC semantic network contains more “steep” association hierarchies, as suggested by Mednick (1962), we would expect that their ability to generate associative responses to target words would be significantly lower than that of the HSC group. Thus, for every target word, we examined the mean amount of unique associations generated by each of the two groups (LSC and HSC), and statistically examined any group difference.

### Procedure

For the creativity measurements, each participant performed the four tasks in a Latin square random order. The CoM task was conducted using the E-prime software (Schneider et al., 2002) and stimuli were presented centrally to the participant on a standard CRT computer screen. Subjects were instructed to recognize whether the two words created a meaningful expression. The RAT was administered as a paper and pencil task. The instructions of the task were presented to the participant and two examples (not used in the task itself) were given. Participants had 15 min to complete the RAT. The TACT was administered as a paper and pencil task. The participants completed each of the TACT sub-tests separately, after they were presented with the instructions for the specific sub-test. Participants had 6 min for each sub-test of the TACT. The RSPM-SV was administered as a paper and pencil task. Instructions of the task were presented to the participant.

The free association generation task was conducted via an in-house Google Application (see De-Deyne and Storms, 2008b for a similar approach). In this application, each target word was presented separately with a clock counting down from a minute and a response box below the target word, where associative responses were entered via the keyboard. Once one minute elapsed, the next target word appeared. All associative responses entered by participants via this application were stored on the Google App Engine Server and were constantly monitored. The 96 target words were divided into four groups of 24 target words per group. Each group of target words was entered separately to our Google application, thus creating four separate sub-applications, each containing a group of 24 target words. Participants were free to complete the association generation task at their own time and computers, They were sent the four applications via a single email and were instructed that each part takes 24 min and that once begun they must complete the whole part without stopping. Furthermore, they were instructed that they could complete the four parts in any order they chose. Only participants that completed all four lists were entered into data analysis. The opening screen of each of the four applications gave the following instructions: “This is an association task. In front of you will appear a single word separately. Please write down as many related responses to this word you can think of. You will have 60 s for each word. For example, for the word *dad* you might write the following responses: mom, son, family, etc.”.

## Results

### Low and high semantic creativity analysis

#### Creativity measures correlation analysis

To examine the relations between the creativity measures, we conducted a correlation analysis between RAT scores, all TACT fluency and quality measures, RSPM-SV scores and all of the CoM measures (response times and accuracy for all four conditions). The full correlation analysis is reported in Supplementary Information Table 1. This analysis did not find any significant correlations between RSPM-SV scores and any of the other creativity measures. The correlation analysis revealed a significant positive correlation between RAT and TACT fluency and quality scores [*r*_(137)_ = 0.22, *p* < 0.008 and *r*_(137)_ = 0.21, *p* < 0.012 for fluency and quality, respectively (two tailed)]. This finding positively relates convergent (RAT) and divergent (TACT) measures of creativity ( i.e., Ward, 1975; Runco and Acar, 2012). The correlation analysis also revealed a negative significant correlation between RAT and CoM response times of novel metaphors [*r*_(137)_ = −0.26, *p* < 0.002 (two tailed)] and a positive significant correlation between TACT quality and CoM accuracy of novel metaphors [*r*_(137)_ = 0.18, *p* < 0.032 (two tailed)]. These two significant correlations replicate findings relating creative ability and novel metaphor processing (Gold et al., 2011).

#### Decision tree analysis

We applied the decision tree approach on the participant's creativity measures data, using the JMP software (www.jmp.com). In our decision tree, participants TACT measures were used as the independent variables and the RAT scores as the dependent variable. Classification rules were derived which compiled various ranges of the different TACT measures in order to predict the classification of specific participants to the different classes of the RAT (25 classes portraying all possible values of the RAT). These classification rules were then sorted from classifying participants with lowest RAT scores to participants with highest RAT scores. Participants positioned in the lower tertile of these classification rules were considered as LSC and participants positioned in the highest tertile as HSC (Table 1). Participants achieving low RAT scores seemed to be classified by having a general low TACT fluency score (<73). They were more specifically classified by various relations between quality and fluency scores in specific sub-tests of the TACT (both verbal and visual). Participants achieving high RAT scores seem to be classified by either having a general high TACT fluency score (≥73) or by having a general low TACT fluency score (<73) combined with low TACT verbal quality scores and high fluency in specific TACT sub-tests (either verbal or visual). Thus, fluency is not a sufficient factor in classifying participants who achieve high RAT scores. Table 1 summarizes the classification rules for both LSC and HSC participants.

**Table 1:** **Classification rules created by the decision tree to classify RAT scores based on TACT measures to LSC (upper panel) and HSC (lower panel) groups**.

**Leaf label**	**Mean**	**Count**
**LSC**		
TACT_F<73&TACT_Verb_Q<15&TACT_1_F<17&TACT_2_F>=10&TACT_4_F<15&TACT_2_F>=13	6.89	9
TACT_F<73&TACT_Verb_Q<15&TACT_1_F>=17&TACT_3_Q<5	8.86	7
TACT_F<73&TACT_Verb_Q>=15&TACT_4_Q<9	5.75	8
TACT_F<73&TACT_Verb_Q>=15&TACT_4_Q>=9&TACT_1_Q>=7	7.11	9
TACT_F<73&TACT_Verb_Q>=15&TACT_4_Q>=9&TACT_1_Q<7	9.50	6
TACT_F<73&TACT_Verb_Q<15&TACT_1_F<17&TACT_2_F<10	6.00	6
**Average**	**7.35**	
**HSC**		
TACT_F>=73&TACT_3_F>=22&TACT_Q>=66	12.09	11
TACT_F>=73&TACT_3_F<22&TACT_F>=76	13.50	12
TACT_F<73&TACT_Verb_Q<15&TACT_1_F>=17&TACT_3_Q>=5	12.13	16
TACT_F<73&TACT_Verb_Q<15&TACT_1_F<17&TACT_2_F>=10&TACT_4_F>=15	10.86	7
**Average**	**12.14**	

*Mean, mean average RAT score of a specific classification rule; count, amount of participants answering to a specific classification rule; Average; average RAT score of the entire groups (LSC, HSC). TACT_1_F, fluency scores of the 1st TACT sub test; TACT_1_Q, quality scores of the 1st TACT sub test; TACT_2_F, fluency scores of the 2nd TACT sub test; TACT_2_Q, quality scores of the 2nd TACT sub test; TACT_3_F, fluency scores of the 3rd TACT sub test; TACT_4_F, fluency scores of the 4th TACT sub test; TACT_4_Q, quality scores of the 4th TACT sub test; TACT_Verb_Q, combined quality scores of the two TACT verbal sub tests (1 and 3); TACT_F, combined fluency scores of all four TACT sub tests; TACT_Q, combined quality scores of all four TACT sub tests*.

To validate this classification to LSC and HSC groups, we examined the difference in performance of the two groups on the CoM task, which has been shown to reliably measure creative ability (Gold et al., 2011; Silvia and Beaty, 2012). An independent samples *t*-test analysis on the difference in CoM scores between LSC and HSC groups revealed that the HSC group had significantly higher accuracy rates and lower average response times in comprehending novel metaphors as compared to the LSC group [*t*_(64)_ = −1.75, *p* < 0.03, η^2^ = 0.07 and *t*_(64)_ = 2.23, *p* < 0.08, η^2^ = 0.05 for response times and accuracy rates respectively (two-tailed)]. Since significant relations have been found between creativity and novel metaphor processing (Gold et al., 2011; Silvia and Beaty, 2012), this analysis validates the decision tree classification to LSC and HSC groups.

#### LSC and HSC group

70 participants (35 LSC and 35 HSC) completed all parts of the free association task. In order to match both groups on the RSPM-SV, from each group two participants with extremely low (less than two standard deviations in the LSC group) or high (more than two standard deviations in the HSC group) RSPM-SV scores were removed. All participants were native Hebrew speakers, with normal or corrected to normal eyesight. Participants received 80 NIS for their participation in the experiment. While the two groups did not significantly differ in any of the demographic details (age, education years, EHI, RSPM-SV), they significantly differed in all creative measures, in the sense that the HSC group had significantly higher scores on all creativity measures (RAT, TACT, CoM-NM) (Table 2).

**Table 2:** **Low Semantic Creative (LSC) and High Semantic Creative (HSC) group details (standard deviations in brackets)**.

	**LSC**	**HSC**
N	33 (13/20)	33 (6/27)
Age	24 (2.4)	23 (2.2)
Education	14 (1.5)	14 (1.4)
EHI	92.5 (9)	90.7 (9.5)
RSPM-SV	111 (8.5)	114 (8.9)
RAT^***^	7 (2.7)	13.2 (3)
TACT F^***^	65.9 (15.7)	88 (24)
TACT Q^***^	34 (12.5)	50.4 (21)
CoM NM-RT^**^	1245 (886)	874 (358)
CoM NM-ACC^*^	0.49 (0.23)	0.6 (0.24)

*N, number of participants comprising each group (male/female in brackets); Age, mean group age in years; Education, mean education years; EHI, mean Edinburgh Handedness Inventory score; RSPM-SV, mean Raven Standard Progressive Matrices Short Version score; RAT, mean Remote Association Test score; TACT F, mean Tel Aviv Creativity Test fluency score; Tel Aviv Creativity Test Q, mean TACT quality score; CoM NM-RT, mean Comprehension of Metaphors Novel Metaphors Response Time; CoM NM-ACC, mean Comprehension of Metaphors Novel Metaphors Accuracy rates*.

* *p < 0.1 for a two-tailed t-test on the difference between groups*;

** *p < 0.05 for a two-tailed t-test on the difference between groups*;

*** *p < 0.001 for a two-tailed t-test on the difference between groups*.

### LSC and HSC network analysis

#### Preprocessing

In order to analyze the data for each group, we first standardized the data into a matrix, in which every column is a different target word and every row is a different association response to a target word. This resulted in a 32,370 (association responses) × 96 (target words) for the LSC group and a 42,367 (association responses) × 96 (target words) for the HSC group.

Since many similar association responses were received for different target words and due to various typing errors within the data, we proceeded to a preprocessing phase in order to construct a matrix where each row was a unique singular association response. This stage entailed two actions—standardizing association responses (i.e., neighbour → neighbor) and converting plural into singular (i.e., fruits → fruit). Next, all standardized association responses were organized into a single matrix and identical association responses were merged using the Minitab software (www.minitab.com). In this matrix, row *i* is a unique association response given by the entire sample, column *j* is a target word and cell(*i*, *j*) denotes the amount of response of associative response *i* to target word *j*. This resulted in a 5557 (unique association responses) × 96 (target words) for the LSC group and a 7617 (unique association responses) × 96 (target words) for the HSC group.

#### Network analysis

**Figure 1:**
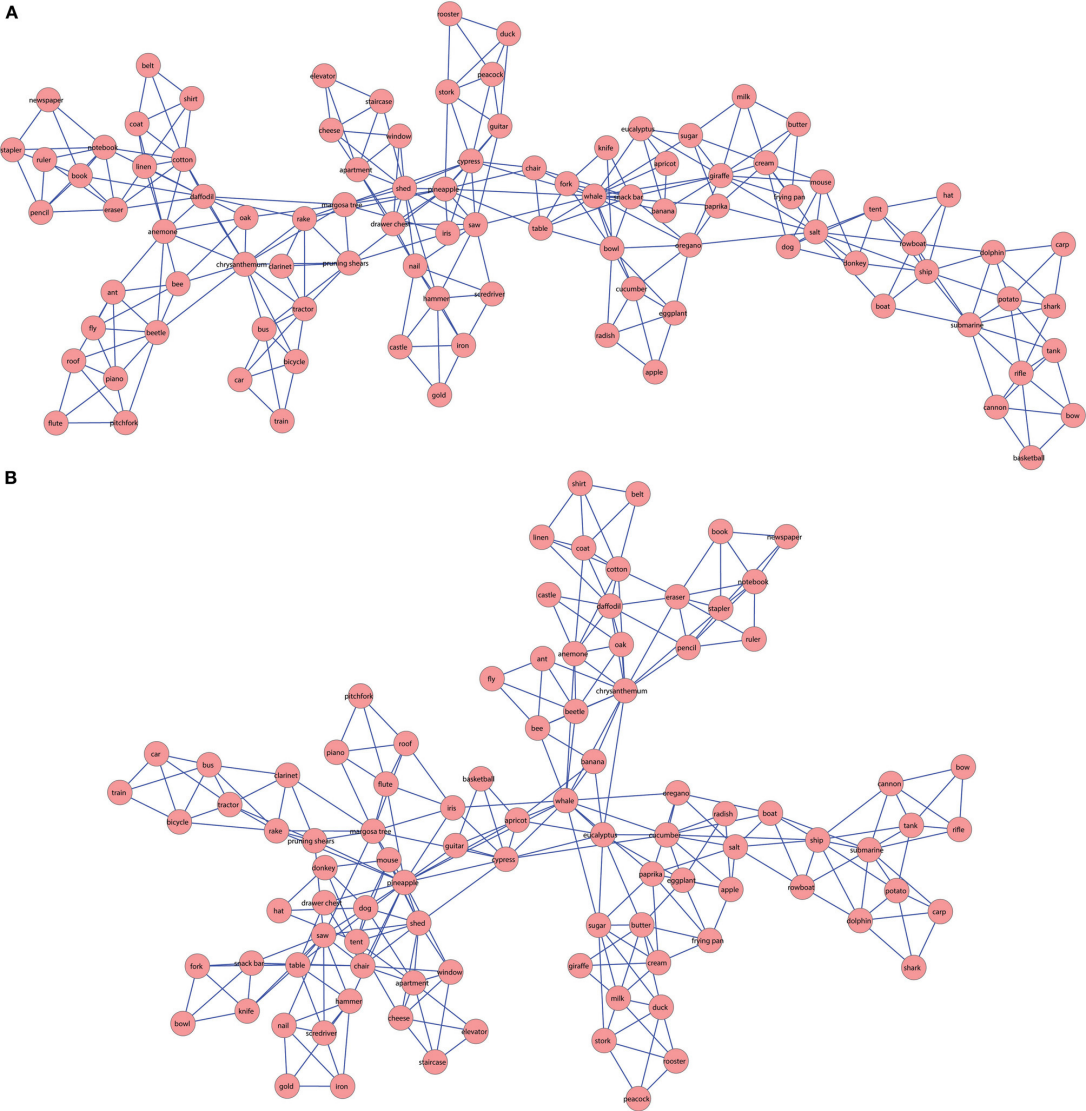
**A 2D visualization of the LSC (A) and HSC (B) semantic networks**. Nodes are the 96 Hebrew target words translated into English. The links between nodes represents an unweighted, undirected connection between nodes.

The association correlations networks were constructed from the association correlation matrices, using the PMFG filtering process (as described in section Association correlation networks). We then calculated different SWN properties of the semantic networks of both groups, to quantitatively examine network differences between them. The values of the different SWN parameters calculated for the LSC and HSC networks are summarized in Table 3. To visualize the network we plotted the graphs using the Cytoscape software (Shannon et al., 2003), and in order to present the Hebrew target words as the labels of the nodes, we translated them into English (Figure 1). In these 2D visualizations of the networks, nodes (words) are marked as red circles and links between them are marked as blue lines. Since these networks are unweighted and undirected, the links merely convey symmetrical relations between two nodes. Both the quantitative analysis of the calculated SWN measures and the qualitative examination of the network visualization reveal differences between LSC and HSC networks. First, the LSC network is more spread out than the HSC network. This is both apparent in the LSC network having a larger ASPL and a larger D than the HSC network. Furthermore, the LSC is less small-worlded than the HSC network, as evident in the S measure. Finally, the LSC network is more modular than the HSC network, as evident in the Q measure. Taken together, these findings indicate that the LSC network is more spread out, less connected and more modular than the HSC network (Table 3).

**Table 3:** **SWN measures calculated for the LSC semantic network and the HSC semantic network**.

**Parameter**	**LSC**	**HSC**
CC	0.67	0.66
ASPL	4.6	3.93
<k>	5.88	5.88
D	12	8
CCrand	0.07	0.06
ASPLrand	2.7	2.7
Q	0.62	0.58
S	6.86	7.76

*CC, clustering coefficient; ASPL, average shortest path length; <k>, average amount of edges a node in the network has; D, diameter; CCrand, Clustering coefficient of random graph; ASPLrand, average shortest path length of random graph; Q, modularity measure; S, small-world-ness measure*.

Next, we conducted the impact analysis to examine any differences between the impact of a specific node between the two networks (LSC and HSC). The impact score for each node for each network was independently calculated as presented above. A Mann–Whitney test analysis on the difference in impact score revealed a significant difference between the two networks [*U*_(192)_ = 3523, *z* = −2.818, *p* < 0.005]. When comparing the amount of negative (impact < 0) and positive (impact ≥ 0) nodes for the two networks an opposing pattern of negative-positive nodes in the two networks is revealed. While in the LSC network there are more negative impact nodes than positive impact nodes, the HSC has more positive impact nodes than negative impact nodes. Furthermore, while the negative-positive impact nodes in the LSC network is more balanced (56–44%), the HSC network has a high percentage of positive impact nodes (65%). This high rate of positive impact nodes might indicate more efficient spread of activation in the network, thus providing another feature characterizing the difference between LSC and HSC networks.

To statistically validate our results, we applied the network validation methods. The simulated random network analysis revealed that for both LSC and HSC networks, all four network measures (CC, ASPL, S, and Q) were statistically significant (all *p*'s < 0.001). Next, we applied the partial bootstrapped analysis. This resulted in a sample distribution of 1000 samples for all measures (CC, ASPL, S, and Q). An independent samples *t*-test was conducted on each network measure to test the difference between the bootstrapped partial networks. These analyses (summarized in Table 4) revealed significant differences between the bootstrapped sample distributions of all measures, indicating that the CC of the partial LSC network was significantly smaller than that of the partial HSC network and the ASPL, S and Q measures of the partial LSC network were significantly larger than that of the partial HSC network (all *p*'s < 0.001). Thus, while these differences were numerically small, they were significantly different and replicated the main finding that the HSC is more small-worlded, more condensed and less modular than the LSC network. These small numerical values probably arise from the partial networks being small.

**Table 4:** **SWN measures calculated for the partial LSC and HSC semantic networks (standard deviations in brackets)**.

**Parameter**	**PLSC**	**PHSC**
CC^***^	0.68 (0.01)	0.69 (0.01)
ASPL^***^	3.19 (0.3)	3.16 (0.3)
S^***^	4.53 (1.05)	4.66 (1.04)
Q^***^	0.55 (0.05)	0.54 (0.05)

*CC, clustering coefficient; ASPL, average shortest path length; S, small-world-ness measure; Q, modularity measure*;

*** *p < 0.001 for a two-tailed t-test on the difference between groups. PLSC, mean partial bootstrapped LSC networks; PHSC, mean partial bootstrapped HSC networks*.

**Figure 2:**
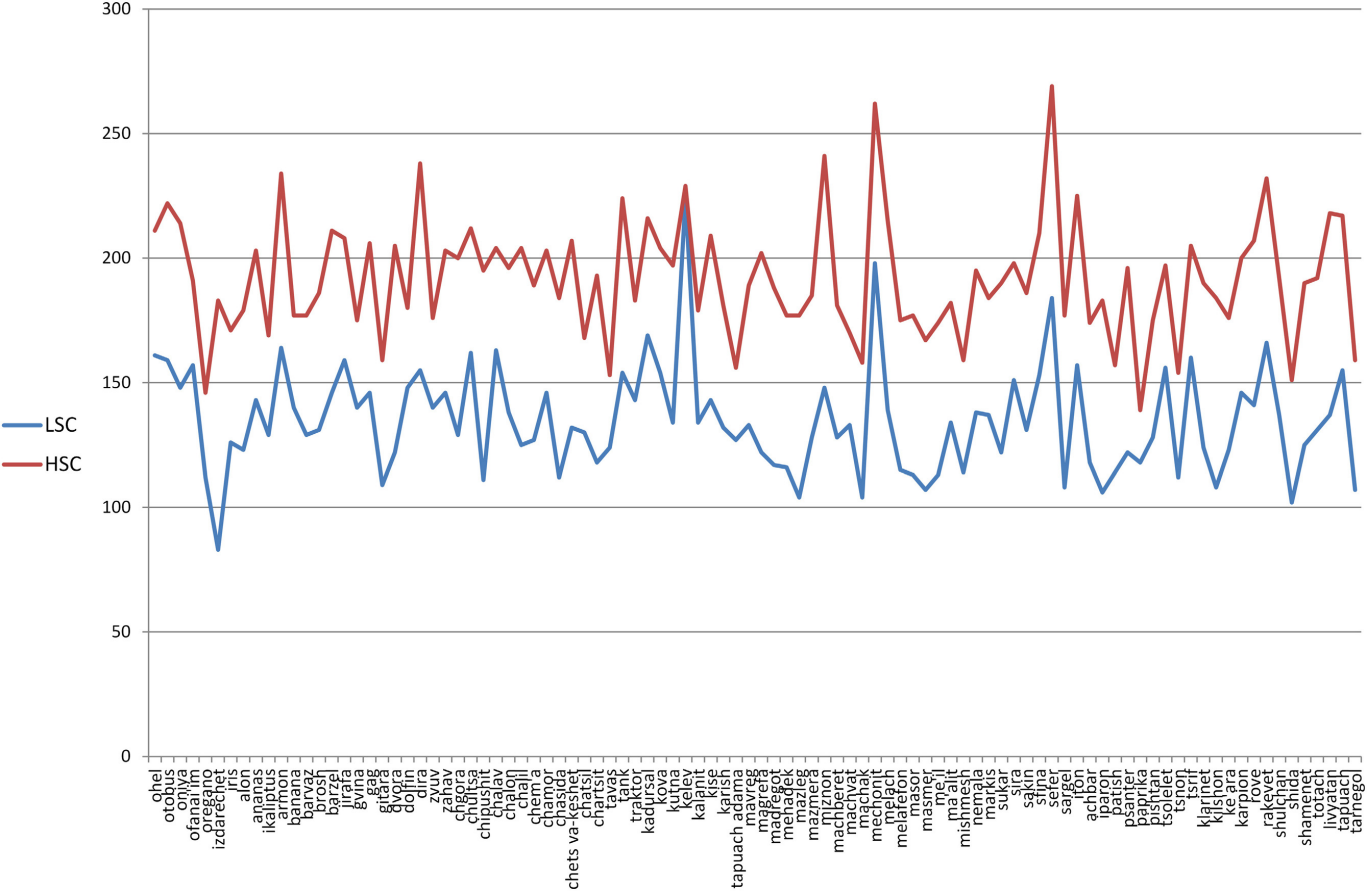
**Average unique association responses generated for target words for the LSC and HSC groups**. X-axis, 96 target words used in the research; Y-axis, amount of mean association responses for a target word. LSC, low semantic creativity group; HSC, high semantic creativity group.

Finally, the difference in association responses generated between the two groups (LSC, HSC) were analyzed by examining the mean amount of unique association responses generated for all target words (Figure 2). As can be seen in Figure 2, the HSC group generated more unique responses than the LSC group for all target words. Despite this difference the amount of unique responses for a specific target word was highly correlated between the two groups [*r*_(190)_ = 0.75, *p* < 0.001]. A One-Way analysis of variance conducted on the effect of group on mean association responses per target word revealed a significant main effect [*F*_(1, 191)_ = 310.937, *p* < 0.001, η ^2^ = 0.614]. A simple effect analysis [corrected for multiple comparisons using the Benjamin-Hochberg correction (Thissen et al., 2002)] was conducted on the average association responses for a given target word between the LSC and HSC groups. This analysis revealed that for 60% of the taget words, there is a significant difference between groups (all *p*'s < 0.01), in the sense that the HSC generated significantly more unique association responses to a target word than the LSC group.

To eliminate any possible associative fluency contamination on network structure, we conducted a network analysis based only on the 10 first associative responses given by a participant to a target word (Benedek and Neubauer, 2013). First, for each group a subset of the raw association responses dataset was comprised, containing the first 10 association responses given to each target word by a specific participant. Next, we extracted the LSC 10 responses and HSC 10 responses semantic networks and examined any possible difference between them. This analysis revealed that the LSC-10 semantic network is less connected, more spread out and less small-worlded than the HSC-10 semantic network (Supplementary Table 2). Thus, the structure of the two networks based on the first 10 responses was similar to the original structure based on all responses. The only network measure which differed was the network modularity, which was lower for the LSC-10 network compared to the HSC-10 network.

## Discussion

In the work presented here, we quantitatively examine the difference in semantic memory network organization between individuals with LSC and HSC ability. A large sample of participants underwent a battery of creativity measures and was classified into LSC and HSC groups based on an objective statistical decision tree approach. Both groups completed a free association paradigm and generated free associations to 96 target words. The similarities between target words based on their free association responses were calculated and used to construct the association correlation matrix separately for each group. These association correlation matrices were used to model the associative networks of both groups, thus representing the organization of the target words in their mental lexicon. This was done to directly investigate, for the first time, Mednick's (1962) theory on individual differences in creativity, by means of network science methodology.

Mednick envisioned the creative process as the combination of remote associations into a novel and appropriate product (Mednick, 1962). He proposed that low creative persons have “steep” compared to “flat” associative hierarchies characterizing more creative persons. Thus, high creative persons may have a more flexible semantic memory organization. Examining the differences between the LSC and HSC networks revealed that the semantic memory network of persons with LSC ability is more spread out (indicated by a higher ASPL), more modular (indicated by a higher modularity measure) and less connected (indicated by a lower small-world-ness measure), than the semantic network of persons with HSC ability. We statistically validated our results by several complementary methods: first, we simulated a large sample of random networks to ascertain that the LSC and HSC network measures calculated did not result from a null-hypothesis random network. Next, we used the bootstrap method (Efron, 1979) to create a large sample of partial LSC and HSC networks and statistically examined the difference between the distribution of networks measures calculated for this large partial networks sample. This analysis found significant differences between the partial-LSC and partial-HSC sample in all network measures examined (CC, ASPL, S, and Q). Finally, we examined the amount of unique association responses generated to each target word by both groups. This analysis revealed a significant difference between groups, in the sense that the HSC group generated significantly more associative responses per target word than the LSC group.

To eliminate any possible associative fluency contamination on the semantic networks, we analyzed the semantic networks of LSC and HSC with only the first 10 associative responses generated by each participant in the group for each of the target words (Benedek and Neubauer, 2013). This analysis verified our general network analysis findings, in the sense that the LSC-10 network is less connected, more spread out and less small-worlded than the HSC-10 network. The only network measure which seems to be affected by associative fluency was the modularity measure, in the sense that the LSC-10 network was less modular than the HSC-10 network. This is in contrast to the results of our analysis of the general networks, which showed that the LSC network was more modular than the HSC network. This higher association fluency for HSC might contribute to the modular structure of the network, leading to more connections between nodes in the network, thus lowering the overall modularity of the network. This is in line with Schilling's theory (2005), that relates creativity to the creation of new links in the network. Future research is required to directly examine the effect of associative fluency on semantic network structure.

The word impact measure (Kenett et al., 2011) was used to examine the effect of each node in both networks. This analysis allows to further examine any general differences between the two networks, but also to examine specifically how each node affects the spread of activation in the network. This is possible as this analysis examines the effect of a node on the ASPL, which is related to spread of activation in the network (Collins and Loftus, 1975; Den-Heyer and Briand, 1986). The null hypothesis for this analysis is a similar effect upon removal of a specific node in both networks. A Mann-Whitney test revealed a significant difference between the impact scores of the two groups. This difference further indicates how the networks differ in their structural properties. Furthermore, a possible dissociation between the percent of positive and negative impact scores between groups was found. In this sense, while the LSC network had a more balanced ratio between negative impact nodes and positive impact nodes (56–44%), the HSC network had a lower ratio of negative impact nodes than positive impact nodes (35–65%). This difference presents another feature which differentiates between the LSC and HSC networks. Possibly, the higher ratio of positive impact nodes in the HSC network facilitates more efficient spread of activation within the network, as removal of these nodes raises the ASPL resulting in the network being further apart.

How can Mednick's theory be related to network measures? first, we argue that Mednick's notion of creativity as a process of connecting remote associations can be measured by a network's small-world-ness nature—the more a network is small worlded, it has a higher CC (connectivity) and a lower ASPL (distance). Thus, a more small-worlded state enables better connectivity within the network, thus better allowing the connection of remote associations and bringing about a creative product (Mednick, 1962; Schilling, 2005). However, a network which is extremely small-worlded may lead to more inappropriate associative relations, thus raising the possibility of semantic chaos (e.g., loose association in schizophrenic states (see Faust and Kenett, under review for a theoretical account of semantic network states). Furthermore, Mednick's theory of “steep” and “flat” associative hierarchies is related to community structure in networks as measured by the network modularity (Newman, 2006; Fortunato, 2010). The modularity measure quantifies the extent to which a network breaks apart into sub-communities, in the sense that the larger it is, the more the network is comprised of sub-communities. Thus, a high modularity score can quantitatively define “steep” associative hierarchies while a low modularity score can quantitatively define “flat” associative hierarchies. To this end, the semantic network of creative individuals needs to be highly connected and contain as small numbers of large association clusters (or “attractor basins;” Rodd et al., 2004; Lerner et al., 2012) as possible (see Cushen and Wiley, 2011, for a recent support of this notion). An extension of Mednick's theory on the difference between the semantic memory structure of low and high creative persons is the spread of the network. Rossman and Fink (2010) have suggestedthat the semantic memory network of more creative persons is more condensed than low creative persons. Our analysis of the structural measures of the LSC and HSC networks empirically verifies this notion (Rossman and Fink, 2010).

How can the features of the semantic network of HSC better facilitate the creative process, including better performance in the RAT? Based on Schilling's (2005) theory that insight is a result of restructuring of the mental lexicon and Griffiths et al. (2007) findings that memory retrieval is similar to the Google search algorithm, we propose that the structure of semantic memory constrains cognitive search processes such as those required in the RAT (Kenett et al., 2011). Once presented with the primed words, the subject activates a search through the semantic network to find the adjoining target word. If the target word is weakly connected or far away from one or more of the primed words, the search process may not have enough activation strength or “get stuck” within a strongly connected module of words surrounding one or more of the primed words. Thus, the search cannot be completed. The successful completion of this search process through the semantic network requires activation of distant associations and creation of new connections within the semantic network (Schilling, 2005), which is more connected, less modular and more condensed.

Recently, Benedek and Neubauer (2013) examined the associative hierarchies of low and high creative participants. This was done by estimating associative hierarchies based on associative strength (relative response frequency). These association strengths were used to map the gradient of associative generation in low and high creative persons which represents their associative hierarchies (see Benedek and Neubauer, 2013 for a full description). The authors did not find any significant difference between the associative hierarchies of low and high creative persons. However, the authors found that the high creative persons differred from the low creative persons in associative fluency and uncommonnes of associations, which are related to each other (Beaty and Silvia, 2012). Thus, the authors concluded that what differentiates between low and high creative persons is not the structure of their associative hierarchies, but rather executive functions required to access semantic content. This approach is in line with increasing literature which shows a tight link between executive functions and creative ability (Nusbaum and Silvia, 2011; Beaty and Silvia, 2012; Benedek et al., 2012a; Silvia et al., 2013). This line of research moves away from a bottom–up (structural) to a top–down (executive functions) difference between low and high creative persons. Nevertheless, the top–down perspective of creativity still recognizes the importance of bottom–up, structural processing in the creative process (Beaty and Silvia, 2012). As Smith et al. (2013) show, the RAT requires a two stage process—a divergent, spreading activation process to generate possible solutions and a convergent, executive process to determine the acceptability of a possible solution. Thus, a full model of the creative process must account for both bottom–up and top–down processing which comprise the creative process. In this regard, network science can provide unique quantitative tools to examine search processes being commenced throughout a semantic memory network. Currently, few attempts have been made at investigating, through a network science perspective, cognitive search processes throughout semantic memory (Goñi et al., 2010; Capitán et al., 2012; Smith et al., 2013). More work is needed to incorporate such work in the study of individual differences in creativity. Thus, the creative process might be envisioned as an efficient search process being commenced upon a semantic memory network. This process is both constrained by the structure of the network and by the efficiency of the search process itself.

A few limitations of this research are related to the small amount of target words comprising the network (96), This is due to the time demanding nature of the paradigm (one minute per word), as larger semantic networks better allow quantitative examinations (Kenett et al., 2011; De-Deyne et al., 2013). Thus, future research is required to investigate larger semantic networks of LSC and HSC groups, to replicate and verify the results presented here. Another limitation due to the method of extracting the semantic networks is that it currently can only represent the network of the entire sample and cannot account for individual semantic networks. Future research is required to expand our network approach to the analysis of individual semantic networks (see Morais et al., 2013 for such a recent novel approach). As individual semantic networks appear to be stable and consistent (Morais et al., 2013), we predict that extracting the semantic networks of individual LSC and HSC persons will replicate the group findings we show in this work. Thus, we do not expect the results found in this research to be due to low consistency between individual semantic networks of participants comprising both groups. Finally, although the LSC network had a higher modularity score than that of the HSC, this difference was small (0.62 compared to 0.58), possibly related to the small amount of words comprising the networks. While this difference was statistically validated via our bootstrapping methodology, further research is required with larger semantic networks of LSC and HSC groups to further examine the modular difference between these two networks. Future work, which we are currently conducting, will empirically examine how the differences we found between the semantic memory structure of low and high creative persons is expressed in behavioral performance and neural activation. Furthermore, more advanced network analysis is in order to further elucidate what differentiates between low and high creative persons from a network perspective. A few examples of such advanced network analyses are dependency network analysis (Kenett et al., 2012), network cascading failures (Buldyrev et al., 2010), and modeling search dynamics in semantic networks.

In summary, we conducted a network science research which quantitatively validates and extends Mednick's (1962) theory on individual differences in creativity. We define Mednick's notion of “flat” and “steep” associative hierarchies in network terms of modularity and show that the semantic network of low creative persons is more modular than that of high creative persons. We also relate his notion of creativity as a process of connecting remote associations to network measures of connectivity, in network terms of small-world-ness state. Finally, we extend his theory and propose the spread of the network as another feature which differentiates between low and high creative persons. Thus, network science allows quantification and examination of classical cognitive theories, such as Mednick's theory of creativity (Mednick, 1962), which were difficult to examine until recently. Analyses of the structure of semantic memory are revelant to several cognitive domains, such as memory, language and high-level cognition and thus network research such as the one presented here is crucial to advancing these fields. Further than investigating and verifying Mednick's theory, we ground semantic creativity with semantic memory structure and cognitive search processes. While we investigate only a specific aspect of creative ability, this work contributes to the expanding neurocognitive empirical investigation of creativity.

### Conflict of interest statement

The authors declare that the research was conducted in the absence of any commercial or financial relationships that could be construed as a potential conflict of interest.
