# Supplementary material for: Investigating the structure of semantic networks in low and high creative persons
Source: Front Hum Neurosci. 2014 Jun 10;8:407. doi: 10.3389/fnhum.2014.00407 (PMC4051268; doi:10.3389/fnhum.2014.00407)
Supplement: Supplementary file 1 [file DataSheet1.DOCX]

**Supplementary Information**

**Supplementary Table 1** – correlations and intercorrelations of the three creativity measures: RAT, TACT and CoM

|  | 1 | 2 | 3 | 4 | 5 | 6 | 7 | 8 | 9 | 10 | 11 | 12 | 13 | 14 | 15 | 16 | 17 | 18 | 19 | 20 | 21 | 22 | 23 |
| --- | --- | --- | --- | --- | --- | --- | --- | --- | --- | --- | --- | --- | --- | --- | --- | --- | --- | --- | --- | --- | --- | --- | --- |
| 1 - Mednick | - | .22^*^ | .18^*^ | .19^*^ | .18^*^ | 0.11 | 0.14 | .23^**^ | .21^*^ | .19^*^ | .19^*^ | .23^**^ | .21^*^ | .22^**^ | .21^*^ | -.3^**^ | -.3^**^ | -.26^**^ | -.21^*^ | .23^**^ | .21^*^ | .12 | .14 |
| 2 – TACT 1_F |  | - | .80^**^ | .49^**^ | .52^**^ | .6^**^ | .55^**^ | .51^**^ | .52^**^ | .9^**^ | .78^**^ | .54^**^ | .55^**^ | .77^**^ | .7^**^ | -.12 | -.15 | -.10 | -.07 | -.05 | -.05 | .11 | -.13 |
| 3 – TACT 1_Q |  |  | - | .42^**^ | .49^**^ | .52^**^ | .47^**^ | .48^**^ | .47^**^ | .74^**^ | .84^**^ | .48^**^ | .52^**^ | .66^**^ | .71^**^ | -.09 | -.16 | -.10 | -.11 | 0 | -.05 | .01 | -.14 |
| 4 – TACT 2_F |  |  |  | - | .89^**^ | .65^**^ | .59^**^ | .72^**^ | .72^**^ | .63^**^ | .59^**^ | .92^**^ | .86^**^ | .85^**^ | .8^**^ | -.11 | -.13 | -.16 | -.15 | .02 | -.04 | .14 | -.07 |
| 5 – TACT 2_Q |  |  |  |  | - | .67^**^ | .66^**^ | .71^**^ | .74^**^ | .66^**^ | .68^**^ | .86^**^ | .93^**^ | .83^**^ | .88^**^ | -.10 | -.14 | -.16 | -.12 | .09 | 0 | .22^*^ | -.14 |
| 6 – TACT 3_F |  |  |  |  |  | - | .81^**^ | .65^**^ | .65^**^ | .89^**^ | .79^**^ | .70^**^ | .71^**^ | .86^**^ | .8^**^ | -.11 | -.16 | -.11 | -.13 | -.02 | -.06 | .06 | -.08 |
| 7 – TACT 3_Q |  |  |  |  |  |  | - | .63^**^ | .68^**^ | .76^**^ | .88^**^ | .66^**^ | .72^**^ | .77^**^ | .85^**^ | -.10 | -.05 | -.08 | -.07 | .03 | -.05 | .15 | -.16 |
| 8 – TACT 4_F |  |  |  |  |  |  |  | - | .94^**^ | .65^**^ | .65^**^ | .94^**^ | .89^**^ | .86^**^ | .84^**^ | -.15 | -.17^*^ | -.12 | -.07 | .03 | -.07 | .10 | -.13 |
| 9 – TACT 4_Q |  |  |  |  |  |  |  |  | - | .65^**^ | .68^**^ | .90^**^ | .94^**^ | .85^**^ | .89^**^ | -.11 | -.11 | -.08 | -.04 | .05 | -.06 | .15 | -.15 |
| 10 – TACT VERB_F |  |  |  |  |  |  |  |  |  | - | .88^**^ | .69^**^ | .70^**^ | .92^**^ | .84^**^ | -.13 | -.17^*^ | -.11 | -.11 | .02 | -.06 | .09 | -.12 |
| 11 – TACT VERB_Q |  |  |  |  |  |  |  |  |  |  | - | .67^**^ | .73^**^ | .84^**^ | .91^**^ | -.11 | -.12 | -.10 | -.10 | .02 | -.06 | .14 | -.18 |
| 12 – TACT VIS_F |  |  |  |  |  |  |  |  |  |  |  | - | .94^**^ | .92^**^ | .88^**^ | -.14 | -.16 | -.15 | -.11 | .03 | -.06 | .13 | -.11 |
| 13 – TACT VIS_Q |  |  |  |  |  |  |  |  |  |  |  |  | - | .9^**^ | .95^**^ | -.11 | -.13 | -.12 | -.08 | .07 | -.03 | .19^*^ | -.16 |
| 14 – TACT F |  |  |  |  |  |  |  |  |  |  |  |  |  | - | .94^**^ | -.15 | -.18^*^ | -.14 | -.12 | .03 | -.07 | .12 | -.12 |
| 15 – TACT Q |  |  |  |  |  |  |  |  |  |  |  |  |  |  | - | -.12 | -.14 | -.12 | -.10 | .05 | -.05 | .18^*^ | -.18^*^ |
| 16 – CM RT |  |  |  |  |  |  |  |  |  |  |  |  |  |  |  | - | .5^**^ | .48^**^ | .45^**^ | -.14 | -.19^*^ | -.04 | -.17^*^ |
| 17 – LIT RT |  |  |  |  |  |  |  |  |  |  |  |  |  |  |  |  | - | .55^**^ | .64^**^ | -.17^*^ | -.38^**^ | .11 | -.31^**^ |
| 18 – NM RT |  |  |  |  |  |  |  |  |  |  |  |  |  |  |  |  |  | - | .74^**^ | -.06 | -.02 | -.04 | -.02 |
| 19 – UR RT |  |  |  |  |  |  |  |  |  |  |  |  |  |  |  |  |  |  | - | .07 | -.08 | .33^**^ | -.27^**^ |
| 20 – CM ACC |  |  |  |  |  |  |  |  |  |  |  |  |  |  |  |  |  |  |  | - | .83^**^ | .39^**^ | .413^**^ |
| 21 – LIT ACC |  |  |  |  |  |  |  |  |  |  |  |  |  |  |  |  |  |  |  |  | - | .11 | .59^**^ |
| 22 – NM ACC |  |  |  |  |  |  |  |  |  |  |  |  |  |  |  |  |  |  |  |  |  | - | -.41^**^ |
| 23 – UR ACC |  |  |  |  |  |  |  |  |  |  |  |  |  |  |  |  |  |  |  |  |  |  | - |

Note – Mednick – RAT scores; TACT 1_F – fluency scores of the 1^st^ TACT sub test; TACT 1_Q – quality scores of the 1^st^ TACT sub test; TACT 2_F – fluency scores of the 2^nd^ TACT sub test; TACT 2_Q – quality scores of the 2^nd^ TACT sub test; TACT 3_F – fluency scores of the 3^rd^ TACT sub test; TACT 3_Q – quality scores of the 3^rd^ TACT sub test; TACT 4_F – fluency scores of the 4^th^ TACT sub test; TACT 4_Q – quality scores of the 4^th^ TACT sub test; TACT VERB_F – combined fluency scores of the two TACT verbal sub tests (1 & 3); TACT VERB_Q – combined quality scores of the two TACT verbal sub tests (1 & 3); TACT VIS_F – combined fluency scores of the two TACT visual sub tests (2 & 4); TACT VIS_Q – combined quality scores of the two TACT visual sub tests (2 & 4); TACT F – combined fluency scores of all four TACT sub tests; TACT Q – combined quality scores of all four TACT sub tests; CM RT – CoM conventional metaphors average reaction times; LIT RT – CoM literal meanings average reaction times; NM RT – CoM novel metaphors average reaction times; UR RT – CoM unrelated average reaction times; CM ACC – CoM conventional metaphors average accuracy ratings; LIT ACC – CoM literal meanings average accuracy ratings; NM ACC – CoM novel metaphors average accuracy ratings; UR ACC – CoM unrelated average accuracy ratings

**Supplementary Table 2** – SWN measures calculated for the LSC 10 responses (LSC-10) and the HSC 10 responses (HSC-10) semantic network. CC – clustering coefficient; ASPL – average shortest path length; <k> - average amount of edges a node in the network has; D – diameter; CCrand – Clustering coefficient of random graph; ASPLrand – average shortest path length of random graph; Q – modularity measure; S – small-world-ness measure.

| **Parameter** | **LSC-10** | **HSC-10** |
| --- | --- | --- |
| **CC** | .67 | .68 |
| **ASPL** | 4.5 | 3.8 |
| **<k>** | 5.88 | 5.88 |
| **D** | 11 | 9 |
| **CCrand** | .07 | .07 |
| **ASPLrand** | 2.7 | 2.7 |
| **Q** | .59 | .64 |
| **S** | 7 | 8.38 |
